# Interplay between Regulatory RNAs and Signal Transduction Systems during Bacterial Infection

**DOI:** 10.3390/genes11101209

**Published:** 2020-10-16

**Authors:** Emma Piattelli, Johann Peltier, Olga Soutourina

**Affiliations:** 1 Institute for Integrative Biology of the Cell (I2BC), CNRS, CEA, Université Paris-Saclay, 91198 Gif-sur-Yvette, France; emma.piattelli@i2bc.paris-saclay.fr (E.P.); johann.peltier@i2bc.paris-saclay.fr (J.P.); 2 Laboratoire Pathogenèses des Bactéries Anaérobies, Institut Pasteur, UMR CNRS 2001, Université de Paris, 75015 Paris, France; 3 Institut Universitaire de France, CEDEX 05, 75231 Paris, France

**Keywords:** small regulatory RNAs, TCS-associated RNAs, stress response, two-component systems, alternative sigma factors, genome-wide approaches, mixed regulatory networks, host adaptation, bacterial pathogens, quorum sensing, community behavior

## Abstract

The ability of pathogenic bacteria to stably infect the host depends on their capacity to respond and adapt to the host environment and on the efficiency of their defensive mechanisms. Bacterial envelope provides a physical barrier protecting against environmental threats. It also constitutes an important sensory interface where numerous sensing systems are located. Signal transduction systems include Two-Component Systems (TCSs) and alternative sigma factors. These systems are able to sense and respond to the ever-changing environment inside the host, altering the bacterial transcriptome to mitigate the impact of the stress. The regulatory networks associated with signal transduction systems comprise small regulatory RNAs (sRNAs) that can be directly involved in the expression of virulence factors. The aim of this review is to describe the importance of TCS- and alternative sigma factor-associated sRNAs in human pathogens during infection. The currently available genome-wide approaches for studies of TCS-regulated sRNAs will be discussed. The differences in the signal transduction mediated by TCSs between bacteria and higher eukaryotes and the specificity of regulatory RNAs for their targets make them appealing targets for discovery of new strategies to fight against multi-resistant bacteria.

## 1. Introduction

Pathogenic and commensal bacteria that colonize mammalian digestive tract or other host locations are subjected to multiple environment fluctuations. Variations in iron and oxygen concentrations, changes in pH and temperature, competition with other bacteria for nutrient availability, host defense, and antibiotic stresses are among the parameters that can affect the life cycle of microorganisms [1,2]. Bacteria have developed different strategies to sense and respond to heterogeneous conditions that lead to the adaptation to the new environment and their survival. A well-coordinated gene expression regulation, including the control of virulence factors, underpins this adaptation [3].

**Figure 1:**
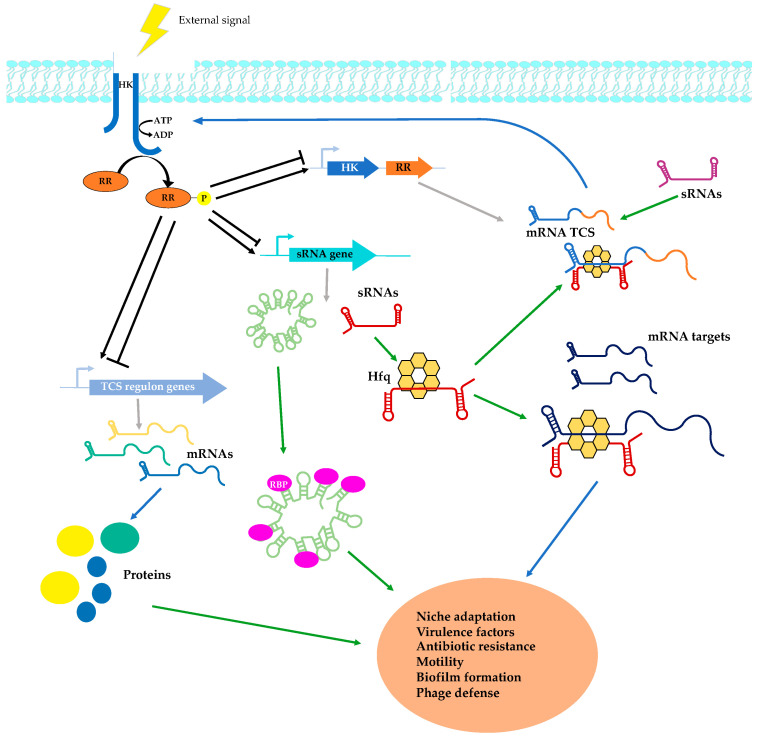
Interplay between regulatory sRNAs and two-component systems during bacterial infection. Bacteria sense external signals through TCSs generally composed of the histidine kinase (HK) sensor and the response regulator (RR). Upon stimulation, the HK autophosphorylates on conserved histidine residues, transferring a phosphate group from the adenosine triphosphate (ATP). The phosphoryl group is then transferred to a conserved aspartate in the receiver domain of the RR. This modification activates the RR that will in turn regulate through its DNA-binding domain the expression of specific genes, activating or repressing (black arrows for activation and bar-headed lines for inhibition) their transcription. Genes regulated by phosphorylated RR encode either proteins or regulatory sRNAs. To mediate their regulatory functions, some sRNAs cooperate with chaperone proteins, such as Hfq, and interact with the mRNA target, altering the expression at post-transcriptional level. sRNAs can also sequester RNA binding proteins (RBPs). Upon detection of specific signals, proteins and sRNAs regulated by TCSs contribute to bacterial infection. Finally, TCSs often autoregulate their expression (genes in blue and orange encoding respectively the HK and the RR), altering the level of specific TCSs in the cell. TCS-encoding genes can also be regulated at the post-transcriptional level by sRNAs comprised or not in the regulon of the corresponding TCS. Grey arrows: transcription, blue arrows: translation.

Small RNAs (sRNAs) are a class of riboregulators that act generally at post-transcriptional level in all kingdoms of life [4]. Bacterial sRNAs and eukaryotic microRNAs (miRNAs) shared many features for target recognition and modulation of gene expression [5,6,7]. In this manuscript, we are focusing on bacterial sRNAs and their importance in the adaptation to a changing environment and in virulence control in pathogens. Bacterial sRNAs are small size, usually non-coding and heterogeneous group of molecules that regulate transcription, translation and mRNA stability through diverse mechanisms of action. sRNAs can bind proteins or effector molecules for riboswitches, interact with foreign DNA or RNA in the case of clustered regularly interspaced short palindromic repeats (CRISPR) RNA, or form a duplex with mRNA target via base-pairing [4,8]. Base-pairing sRNAs are important post-transcriptional regulators that can either enhance or repress mRNA decay and/or translation by binding in most cases to the 5′ untranslated region (5’ UTR) of mRNA. Based on their location with respect to the target, sRNAs are differentiated between *cis*- and *trans*-encoded, which could act either in *cis* or in *trans* [7,9]. While *cis*-encoded sRNAs bind the target with perfect complementarity, *trans*-encoded sRNAs are only partially complementary to their targets and usually require the well-characterized RNA chaperone protein Hfq or ProQ for their action in Gram-negative bacteria [10,11]. The role of Hfq in Gram-positive bacteria is less defined and *trans*-encoded RNAs may recognize their targets in an Hfq-independent manner [12]. Transcription of most known *trans*-encoded sRNAs is controlled by transcriptional factors that include Two-Component Systems (TCSs) and alternative sigma factors. TCSs allow adjusting bacterial gene expression in response to environmental cues. Many connections between TCSs and sRNAs have recently been unraveled and it is becoming clear that they form complex regulatory networks in bacteria (Figure 1). Well-documented examples in both Gram-positive and Gram-negative bacteria illustrate this interplay between TCSs and sRNAs. For instance, porin-dependent membrane permeability and quorum-sensing control of pathogenicity involve TCS-regulated sRNAs together with feedback regulatory loops. Several review articles cover various aspects of this large subject [13,14,15,16]. In this manuscript, we are reviewing the most recent advances in the characterization of the role of sRNAs related with virulence control in major human pathogens that are connected with TCSs or stress-related sigma factors in complex regulatory circuits. The main approaches for the identification and analysis of these sRNAs will also be discussed as well as perspectives for future applications.

## 2. sRNAs Regulating Host-Pathogen Interactions

Crucial steps in the pathogen infection cycle include the capacity to limit and repair damages during host-related stress conditions, to escape the immunity system and to develop antibiotic resistance [17,18,19]. The regulation of all these steps during host colonization and infection is tightly regulated in pathogens, and sRNAs are important actors in these regulatory processes [16]. Indeed, regulatory events mediated by sRNAs are intimately connected to the transcriptional bacterial network [13]. The expression of many sRNAs is controlled by TCSs (Table 1) and specific stress-responsive alternative sigma factors [13,14,20]. Alternative sigma factors influence the promoter specificity of the core RNA polymerase to direct selective transcription of different gene sets, providing an efficient way for bacteria to rapidly adapt to various environmental changes. The number of sigma factors encoded by different bacterial species varies considerably and generally relates to the diversity of environmental conditions encountered by the organism [21]. Classical TCSs consist of a sensor histidine kinase (HK) and a related response regulator (RR). The sensing of the environmental signal by the HK activates the autokinase domain of the HK, causing the transfer of a phosphoryl group from adenosine triphosphate (ATP) to a conserved histidine residue. The phosphoryl group is then transferred to a conserved aspartate in the receiver domain of the RR. An effector or output domain is present in the RR together with the receiver domain. This domain senses the conformational change induced by the phosphorylation of the aspartate and elicits the specific response [22]. The majority of effector domains have DNA-binding activity and function to activate or repress transcription of specific genes [23]. Most HKs are bifunctional enzymes also displaying phosphatase activity to modulate the phosphorylation level of their cognate RR [23]. Many TCSs contain sRNAs in their regulon, resulting in a fine-tuned gene expression [14]. Similarly to sigma factors, it has been established that the abundance of TCS signaling genes correlates with the lifecycle of bacteria. Specifically, bacteria that colonize multiple habitats usually encode a larger number of these signaling and regulatory proteins, enabling an efficient sensing and response to fluctuations of environment. In contrast, bacteria that primarily live in a uniform habitat encode relatively few TCS signaling genes [24].

### 2.1. sRNAs Involved in Stress Response

Bacteria encounter numerous stresses when they enter in contact with the host [98]. The envelope is a physical barrier that protects against environmental stresses and constitutes a crucial interface for host–pathogen interactions. To adapt to the host environment, bacteria have developed general stress responses and Envelope Stress Responses (ESR) [17,99]. The alternative sigma factors σ^S^ or σ^B^ orchestrate the general stress reponse in Gram-negative bacteria and Gram-positive bacteria respectively [99], while ERSs are mediated by the alternative sigma factor σ^E^ and several TCSs including PhoPQ and EnvZ/OmpR. In this section, we will present new sRNAs present in stress-associated regulatory networks and we will discuss such regulations for major human pathogens.

#### 2.1.1. sRNAs Regulated by Alternative σ-Factors

The alternative sigma factor σ^B^ modulates the general stress response of several Gram-positive bacteria. This sigma factor is responsible for the transcription of genes that confer stress resistance to the vegetative cell and is important for virulence [100]. In *Staphylococcus aureus*, the transcription of the sRNA RsaA is σ^B^-dependent [101]. RsaA activates the production of biofilm and inhibits the capsule synthesis through the repression of the master regulator MgrA translation. RsaA base-pairs with *mgrA* mRNA in two different regions: one next to the ribosome-binding site (RBS) and the other in the coding sequence [102]. The duplex formation leads to the sequestration of the RBS and to the recruitment of RNase III to degrade the duplex. Although MgrA is the main target of RsaA, other targets, such as mRNAs for cell wall hydrolases and FLIPr, a secreted immunomodulatory molecule, have been identified [103]. Using mouse infection models, RsaA was shown to diminish invasiveness but enhance local colonization, favoring chronic infection [102].

In *Listeria monocytogenes*, expression of at least two sRNAs, SbrA and Rli33-1, is directly controlled by σ^B^ [104,105]. While the function of SbrA remains to be investigated, Rli33-1 plays a role during infection [106]. Rli33-1 sRNA belongs to the multicopy family LhrC, encompassing 7 members [77]. Besides Rli33-1, LhrC sRNAs, comprising LhrC1-5 and Rli22, are positively regulated by the TCS LisRK that responds to cell envelope stress [71,72,76,77]. Transcription of all LhrC sRNAs except Rli22 is strongly induced during intracellular bacterial growth in murine macrophages. In addition, a deletion mutant of Rli33-1 is affected in its survival in murine macrophages and is less virulent in *Galleria mellonella* and murine infection models [106]. All LhrC sRNAs are also highly induced when *L. monocytogenes* is exposed to human blood, and the expression of members LhrC1–5 is increased in the presence of excess heme, the core component of hemoglobin [76,107]. The LhrC family of sRNAs negatively regulates the same mRNA targets through base-pairing to the RBS region, resulting in inhibition of translation or a decrease of mRNA stability [72,74,75,77]. LhrC targets include three surface-exposed proteins involved in virulence, the adhesin LapB, the oligopeptide-binding protein OppA and the CD4^+^ T cell-stimulating antigen TcsA. In addition, strong evidence suggests that the heme-binding proteins Hbp1 and Hbp2, and the heme oxygenase-like protein Lmo0484 involved in heme uptake and utilization are other members of the LhrC regulon, further reinforcing a link between the response to heme toxicity and cell envelope stress in *L. monocytogenes* [76].

In *Escherichia coli*, several sRNAs are involved in the regulatory network of the general stress response sigma factor σ^S^ (for a detailed review on the general stress response mediated by σ^S^ in bacteria see [108]).

Unassembled outer membrane proteins (OMPs) and lipopolysaccharides (LPS) damages caused by physical and chemical stresses generally trigger the ESR σ^E^-dependent response in Gram-negative bacteria [109]. Upon stimulation, the activation of σ^E^ is mediated by a regulated inter-membrane proteolysis (RIP) [110] that results in the cleavage of the inhibitor anti-σ factor RseA and the release of σ^E^ in the cytoplasm. σ^E^, as a cofactor of RNA polymerase, recognizes specific promoters and initiates the transcription of around 100 genes implicated in outer membrane (OM) modifications and repair [100].

In *Salmonella* and *E. coli*, σ^E^-regulated sRNAs repress protein synthesis of all of the most abundant OMPs. The strongest promoters recognized by σ^E^ drive the transcription of two sRNAs: MicA and RybB [111]. These two Hfq-dependent sRNAs act as posttranscriptional repressors and bind with a limited base-pairing the 5’ UTR of target mRNAs, altering their translation and stability [112]. MicA and RybB both repress the synthesis of several major OMPs to limit the production of these components when the OM is damaged [112,113]. In *E. coli*, deletion of *rpoE*, the gene encoding σ^E^, is lethal [114], but it has been shown that MicA and RybB can rescue the cells from lethality associated with loss of σ^E^ activity [112]. Thus, an adequate activation of these two sRNAs is essential and the promoter strength of *micA* and *rybB* underlies their physiological importance in balancing the OM during stress conditions. Interestingly, these two sRNAs share the regulation of some targets, such as *ompA*, in non-overlapping regions, suggesting that they might need to cooperate to achieve optimal repression [112].

Besides MicA and RybB, σ^E^ regulates an additional Hfq-dependent sRNA, MicL in *E. coli* and *Salmonella*. This sRNA inhibits the expression of the most abundant OM lipoprotein Lpp by specifically targeting the corresponding mRNA, which tethers the OM to the peptidoglycan layer [115].

In *Vibrio cholerae,* σ^E^ activates the expression of two other Hfq-dependent sRNAs, VrrA and MicV. VrrA recognizes the 5’ region of the *ompA* mRNA, repressing its translation and causing increased release of OM vesicles (OMVs). OMVs are important for niche colonization, transport of virulence factors into host cells, modulation of host defense, and response and communication with the surrounding environment. Accordingly, a *vrrA* mutant showed increased ability to colonize the small intestine of mice as compared with the wild type [116,117]. VrrA also represses the major OMP OmpT, the biofilm matrix protein RbmC and the ribosome-binding protein Vrp, involved in starvation survival [118,119,120]. The second sRNA, MicV, shares the same seed-pairing domain as VrrA and more than 23 genes are targeted by both sRNAs including the genes encoding OMP OmpA and OmpT, suggesting a redundant function of VrrA and MicV [121].

#### 2.1.2. Other ESR-Regulated sRNAs

The PhoPQ TCS is another transduction system that responds to cell envelope damage and significantly remodels the envelope. In *Salmonella enterica* serovar Typhimurium, PhoPQ is a major regulator of virulence and is activated by exposure to low pH, divalent cations such as Mg^2+^ and Ca^2+^, and cationic antimicrobial peptides (CAMPs) [122]. Genes encoding OMPs, IM transporters that buffer the cytosolic pH and enzymes for the modification of the LPS are among those positively regulated by the activated PhoP (see review [122] for further details). The remodeling of LPS alters the interaction with CAMPs, providing resistance against these molecules [123,124,125]. In the regulatory control of the LPS, the PhoPQ TCS activates the expression of the MgrR sRNA. Specifically, this Hfq-dependent sRNA inhibits the synthesis of the LPS phosphoethanolamine transferase EptB by binding the 5’ UTR of the corresponding mRNA. Deletion of the MgrR sRNA results in expression of *eptB* and is associated with a higher resistance to the CAMP Polimixin B [38].

PhoPQ also induces the transcription of the Hfq-binding sRNA PinT, which is involved in the regulation of the expression of genes located in the pathogenicity island 1 (SPI-1) of *Salmonella* [41,42,43]. Type III secretion system (T3SS) encoded by SPI-1 is important for the invasion of macrophages and intestinal epithelial cells. Three regulatory proteins, HilD, HilC, and RtsA activate the transcription of the *hilA* gene, encoding the transcriptional activator of the structural and primary effector genes of the SPI-1 T3SS apparatus. Once inside the phagosome of the eukaryotic cells and during systemic infection, genes required for intracellular survival are expressed while the SPI-1 gene expression is shut off. The PhoPQ TCS inhibits SPI-1 gene expression by directly repressing *hilA* transcription. In addition, the sRNA PinT inhibits *hilA* and *rtsA* translation. PinT base-pairs with a region close to the AUG translation initiation codon of *hilA* and *rstA* mRNAs. This interaction blocks translation of *hilA* and *rstA* mRNA by inhibiting the ribosome binding and triggers the degradation of the *rtsABCD* polycistronic mRNA by the RNA degradosome [42]. Besides its regulatory role on SPI-1 gene expression, PinT directly represses the translation of *ssrB*, encoding the primary regulator of the pathogenicity islands 2 (SPI-2) T3SS and inhibits motility by repressing *crp*, a cAMP receptor protein-encoding gene involved in the activation of the flagellar master regulatory genes *flhDC* [42]. Importantly, a *pinT* mutant outcompetes the wild-type strain during systemic stages of infection in competition assays [42].

In addition to MgrR and PinT, PhoPQ also regulates the expression of a *cis*-encoded long antisense RNA named AmgR that is transcribed from an intergenic region located between the *mgtC-mgtB* genes within the *mgtCBR* polycistronic messenger. MgtC is an inner membrane protein critical for intramacrophage survival and mouse virulence and involved in growth in low Mg^2+^ conditions; MgtB is a Mg^2+^ transporter; and MgtR is a short peptide regulator that binds MgtC, promoting MgtC degradation by the protease FtsH [126]. The AmgR transcription reduces the MgtC and MgtB protein levels in an Hfq-independent manner requiring the RNAse E. Conversely, *amgR* deletion increases the level of the same proteins, enhancing bacterial virulence [40]. AmgR is also required for replication in a low Mg^2+^ environment [40]. Interestingly, expression of AmgR and its target mRNA is controlled by the same regulator since PhoP-P also recognizes and activates the transcription from *mgtCBR* promoter. Thus, two negative regulators of MgtC, AmgR and MgtR, are expressed under the same conditions. Nevertheless, they make different contributions to *Salmonella* virulence as an *amgR* promoter mutant is hypervirulent in mice, unlike a *mgtR* deletion mutant [40,126]. Thus, AmgR plays an important role in the survival of *Salmonella* within macrophages and in its virulence in mice [40,127].

High osmolarity in the intestinal lumen of the host is an important stress faced by gut pathogens. In *E. coli*, the EnvZ/OmpR TCS senses osmotic stresses and differently regulates the synthesis of OmpF and OmpC, the two major porins that facilitate the diffusion of small hydrophilic solutes across the OM [128]. When osmolarity increases, OmpR activates the expression of the sRNA MicF. MicF prevents the translation initiation of *ompF* by direct base-pairing to a region of the *ompF* mRNA encompassing both the RBS and the start codon [36]. The repression of OmpF contributes to the protection of the cell during high osmolarity conditions. Indeed, the pore diameter of OmpF is larger than that of OmpC and when expressed is responsible for a faster diffusion rate of osmolytes [129]. Porins mediate the passive diffusion of antibiotics across the OM and are associated with antibiotic resistance in the Gram-negative bacteria. In line with this notion, overexpression of MicF increases *E coli* resistance to different antibiotics, including cephalosporins, while MicF depletion has an opposite effect [130,131].

The EnvZ/OmpR regulon includes two other Hfq-dependent sRNAs, OmrA and OmrB, involved in the regulation of the OM composition. These two sRNAs are positively regulated by OmpR and are redundant on most targets, using their almost identical 5′ tails for base-pairing [32]. Proteins repressed by OmrA and OmrB include many OMPs such as the protease OmpT and the iron transporters CirA, FecA, and FepA. To date, the functional link between the iron transport inhibition and the EnvZ/OmpR activation remains unclear [31].

The sRNAs OmrA/B also play important roles in the regulation of the flagellar synthesis and biofilm formation by targeting master regulatory genes. Bacterial flagellum synthesis genes form an ordered cascade in which the expression of one gene at a given level requires the transcription of another gene at a higher level. The flagellar synthesis master regulator FlhDC is encoded in the class I operon and activates the transcription of the class II sigma factor *fliA* (σ^28^) and anti-sigma factor *flgM*. FliA is held inactive through direct protein interaction with FlgM and directs expression of the class III genes upon secretion of FlgM [132]. OmrA and OmrB base-pair with the 5′ UTR of *flhDC* and the early coding region of *flgM*, inhibiting translation in both cases [33,34]. Thus, OmrA/B repress the initial step of the flagellar pathway via FlhDC, but also activate FliA and the subsequent class III gene expression via FlgM repression. OmrA/B also target the transcript of the master regulator of biofilm formation CsgD [33], inhibiting *csgD* gene expression [133,134]. Interestingly, OmrA/B post-transcriptionally regulate the expression of *ompR* by base-pairing with its 5′ UTR, forming a negative feedback regulatory loop [31,32].

In *V. cholerae*, high osmolarity and acid stress induce the expression of the CoaR sRNA via the EnvZ/OmpR TCS. CoaR targets the *tcpI* gene encoding the negative regulator of the major pilin subunit TcpA, which is important for host colonization. Accordingly, the deletion of this sRNA negatively impacts the colonization of the small intestine of mice [58,59].

### 2.2. TCS-Associated sRNAs Involved in Quorum-Sensing Signaling

Quorum sensing is a signaling process that allows community-wide coordination and collective behaviors in bacteria. The signaling molecules activating the quorum sensing are the extracellular autoinducers (AIs). The concentration of AIs is proportional to the bacterial cell density. Once activated, quorum sensing signaling modulates gene expression [135]. Important virulence-associated sRNAs participate in this regulatory network [135]. The switching to the community behavior, in some pathogens, includes the induction of virulence phenotype [136].

In *S. aureus*, the quorum sensing signaling is regulated by the *agr* locus, which includes the well characterized sRNA RNAIII. This locus comprises two open reading frames (ORFs) whose transcription is initiated from two promoters named P2 and P3 that are located in divergent orientations in the chromosome [82]. The P2 promoter regulates the transcription of the polycistronic RNA, RNAII, encoding the AgrBDCA proteins. AgrB and AgrD are responsible for the synthesis, maturation and secretion of the AI peptide (AIP) while AgrC and AgrA form a TCS [82]. AIP, which serves as an indicator of the local population density, binds to the sensor kinase AgrC that in turn activates AgrA by phosphorylation. AgrA-P recognizes sites in the P2 and P3 promoters, activating the transcription of RNAII and RNAIII, respectively [82].

The 514 nt sRNA RNAIII is one of the main intracellular effectors of the *agr* system and plays a key role in the virulence of *S. aureus* by controlling the switch between early expression of surface proteins and late expression of several exotoxins [81]. Interestingly, RNAIII acts as a dual function RNA since it also encodes in its 5’ end a small toxin, the phenol-soluble modulin (PSM) δ-hemolysin, that confers hemolytic activity to the bacterium [82]. RNAIII is a very structured sRNA with 14 stem loops involved in base pairing-dependent regulation of mRNA targets [137]. To date, many direct RNAIII targets have been identified, including two transcriptional regulators by means of which RNAIII indirectly modulates the transcription of many secondary targets [82]. It should be noted that the interaction between RNAIII and its direct mRNA targets does not require the RNA chaperone protein Hfq [138]. RNAIII acts both as a repressor and an activator of translation. Target repression is achieved through inhibition of ribosome binding, thus preventing translation initiation and frequently stimulating the specific recognition and degradation by RNase III [139]. RNAIII also stimulates translation of three mRNA targets for the global regulatory protein MgrA, the α-hemolysin Hla and the extracellular adherence protein Map. Binding of both the 5’ and the 3’ end of RNAIII to the 5’ UTR of MgrA mRNA leads to the stabilization of the MgrA transcript and enhances MgrA production. This regulator acts as a repressor of biofilm formation and cell autolysis and as an activator of the *agr* locus and capsule gene expression [82,140]. The mechanism for Hla activation is more original with the binding of RNAIII to the 5’ UTR of *hla,* releasing the RBS, which is otherwise sequestered by intramolecular base-pairing [141]. Activation of Map by RNAIII might be mediated by a similar mechanism but has not been studied in detail [142].

RNAIII is a posttranscriptional regulator of multiple virulence genes that plays a key role in the late stages of infection by mediating the switch between adhesion and invasion [143]. In line with this statement, many clinical isolates from acute infection express RNAIII [144]. Targets whose translation is repressed by RNAIII are implicated in the early stages of infection and include surface proteins important for the adhesion and immune evasion (protein A encoded by *spa* [145], coagulase encoded by *coa*, immunoglobin-binding protein encoded by *sbi* [146], and fibrinogen-binding protein SA1000 [143]). Conversely, RNAIII activates synthesis of exotoxins both directly (induction of δ-hemolysin and α-hemolysin synthesis) and indirectly via the negative regulation of Rot, a transcriptional factor repressing the expression of several toxins [143].

Interestingly, *agr* defective strains have been isolated from patients colonized by *S. aureus* [147,148]. These isolates have lost the ability to disseminate in the host but are associated with persistent bacteremia and are thought to be positively selected during chronic infection and dormant states, a situation associated with biofilm formation [16,82].

In *S. aureus*, AgrA also activates the transcription of psm-mec, a dual function RNA that encodes the 22 amino-acid PSM-mec cytolysin [149] and directly interacts with *agrA* mRNA, inhibiting *agrA* translation. AgrA repression leads to a decrease of toxin production and the attenuation of virulence in mice [150]. Interestingly, psm-mec, which is transcribed from the SCC*mec* mobile genetic element, is present in the hospital associated-methicillin resistant *S. aureus* but absent in the more virulent community-acquired methicillin-resistant *S. aureus*, suggesting a correlation between high virulence and absence of psm-mec [150].

Finally, the AgrAC TCS of *S. aureus* represses the expression of an sRNA designated ArtR [83]. ArtR directly binds, in an Hfq-independent manner, to the 5’ UTR of *sarT* mRNA, thus promoting the duplex degradation by RNase III. SarT is a repressor of the α-hemolysin virulence factor Hla and, therefore, ArtR indirectly activates the expression of *hla*.

The *agr* locus is also present in the genome of other Gram-positive bacteria such as *Enterococcus faecalis*, *L. monocytogenes*, *Clostridium botulinum,* and *Clostridioides difficile* [151,152,153,154]. An *agr* deletion in these bacteria is associated with a decrease of the virulence, but no sRNAs have been found yet in their regulon [151,152,153,154]. In *Clostridium perfringens*, the *agr*-like operon does not contain a TCS but it has been suggested that VirS/VirR could serve this function instead [155]. Interestingly, the VirS/VirR regulon comprises an important sRNA, VR-RNA [93]. This 386 nt sRNA is involved in the regulation of 147 genes including the α-toxin (or phospholipase C) encoded by *plc* and the κ-toxin (or collagenase) encoded by *colA* [94,95]. The mechanism of activation of *colA* translation by VR-RNA has been investigated [96]. VR-RNA base-pairs with the 5’ UTR of *colA* mRNA, leading to the cleavage of this extremity and to the subsequent stabilization of *colA* mRNA.

### 2.3. TCS-Associated sRNAs Involved in CRISPR-Cas Immunity and Competence Control

During infection, pathogens need to cope with the presence of genetic invaders like bacteriophages and other sources of foreign nucleic acids. The bacterial life-style within high cell density communities favors viral predation and genetic exchanges through horizontal gene transfer (HGT) [156]. Depending on the nature of the new genetic information, the outcome of its uptake can be either deleterious or beneficial for bacterial survival in host environments. For this reason, the efficiency of protective mechanisms and the acquisition of new genetic elements need to be tightly balanced. Bacteria have developed sophisticated mechanisms for these two antagonistic functions of defense against genetic invaders and HGT. Thus, the regulation of defense systems including CRISPR-Cas on the one hand and new DNA acquisition mechanisms including natural competence systems on the other hand is extremely important for bacterial adaptation to changing environments with fitness cost considerations. Recent studies provided new examples of such regulatory mechanisms involving sRNAs under the control of TCS in major pathogens [64,157,158,159,160,161].

Together with many community-level behaviors, quorum sensing has been recently shown to control CRISPR-Cas-mediated immunity in bacteria, including pathogens [157,158,159]. The regulatory components of the LuxIR-like TCS quorum sensing system in Gram-negative bacteria, named SmaIR in *Serratia* and LasIR/RhlIR in *Pseudomonas aeruginosa,* induce the expression of *cas* operons and CRISPR arrays of multiple CRISPR-Cas systems of type I-E, I-F and III-A, thus affecting both interference and immunity acquisition functions of CRISPR-Cas [157,158]. The link between quorum sensing and CRISPR-Cas regulation was recently further confirmed by studies in *P. aeruginosa* clinical strains, demonstrating the potential of a quorum quenching enzyme degrading quorum-sensing signaling molecules to modulate CRISPR-Cas expression [159]. Quorum sensing was shown to act synergistically with another cue, low environmental temperature associated with slow growth, to control CRISPR-Cas function in this pathogen [162].

In addition to adaptive immunity, noncanonical functions have been suggested for the components of CRISPR-Cas systems (Cas proteins, crRNAs and other CRISPR-associated RNAs) that are implicated in the control of bacterial pathogenesis and physiology [163,164,165,166,167]. The molecular mechanisms of CRISPR-Cas-mediated control of gene expression remain in many cases to be explored, but important links of these regulatory processes with virulence, biofilm formation and envelope stress response could be emphasized in several pathogens, i.e., *Francisella novicida*, *Neisseria meningitidis, Campylobacter jejuni, Legionella pneumophila*, *L. monocytogenes,* and *P. aeruginosa* [164,166,168]. Interestingly, a recent work suggests targeting of quorum-sensing systems by CRISPR-Cas in *Salmonella* to mediate virulence and biofilm formation regulation [168].

Besides quorum-sensing, recent studies revealed the role of other TCS in the regulation of CRISPR-Cas systems [64,160]. In *P. aeruginosa,* KinB-AlgB TCS, involved in extracellular polysaccharide alginate biosynthesis, has been identified as a regulator of expression and activity of type I-F CRISPR-Cas [64]. This work provides an example of how a CRISPR-Cas system is controlled during lifestyle transitions in this opportunistic pathogen. In the cariogenic bacterium *Streptococcus mutans*, VicR/K, a TCS important for biofilm formation, competence, stress tolerance and bacteriocin production, is implicated in the control of type I-C and type II-A CRISPR-Cas to modulate stress response, DNA repair and natural transformation [160].

Despite the crucial importance of defense against mobile genetic elements, HTG provides a powerful mechanism to acquire new traits contributing to bacterial adaptation and survival. An important human pathogen, *Streptococcus pneumoniae,* is able to uptake new DNA through natural competence induction. Several sRNAs are implicated in the competence control of this bacterium [161,169,170,171]. This includes the sRNA, srn206, which is essential for competence initiation and targets the *comD* gene, encoding the histidine kinase of the ComDE TCS [170]. Five sRNAs of 87–151 nt in length named csRNA1-5 (for cia-dependent small RNAs), sharing high sequence similarity, have been identified as members of CiaRH TCS regulon [161,171]. This TCS, highly conserved among streptococci, affects β-lactam resistance, autolysis, virulence, and competence development. The competence control is mediated by five csRNAs whose expression is driven by the five strongest promoters within the CiaRH regulon. Target predictions and translational-fusion analysis identified the *comC* gene, encoding the precursor of competence-stimulating peptide, as a target of csRNAs [171]. By inhibiting *comC* translation, csRNA could block the competence initiation process linking CiaRH to competence control.

### 2.4. Signal Transduction Systems-Associated Riboswitches

Riboswitches are highly structured *cis*-acting elements located at the 5’ UTR of mRNAs that are involved in the regulation of gene expression upon binding of specific molecules (reviewed in [172]). Currently, almost 40 distinct classes of riboswitches binding diverse ligands such as metabolites or ions have been identified [173]. While several riboswitches have been shown to contribute to bacterial pathogenicity (reviewed in [174]), only a few are associated with TCS or specific sigma factors. Examples are developed below.

Cyclic diguanylate monophosphate (c-di-GMP) is a second messenger in bacterial systems and a key feature in the control of critical lifestyle choices, such as the transition between planktonic and biofilm growth [175]. Elevated levels of c-di-GMP typically promote sessile lifestyles such as biofilm formation; in contrast, low levels of c-di-GMP are associated with motility [175]. *C. difficile* 630 encodes 18 predicted diguanylate cyclases and 19 predicted phosphodiesterases, many of which have confirmed enzymatic activity [176]. In addition, *C. difficile* 630 encodes 16 class I and class II c-di-GMP sensing riboswitches [177], underlining the importance of c-di-GMP signaling in this human pathogen. The *flgB* operon, encoding flagellar genes and the flagellar sigma factor σ^D^, is preceded by the Cdi1_3 c-di-GMP-responsive riboswitch in *C. difficile* [177,178,179]. C-di-GMP binding to Cdi1_3 in the 5′ UTR of the *flgB* operon mRNA causes premature termination of transcription and consequently leads to a decrease in the transcription of the *flgB* operon [177,178,179]. Interestingly, σ^D^ positively controls TcdR, an alternative sigma factor that directs transcription of the toxin-encoding genes *tcdA* and *tcdB* for the main virulence factors of *C. difficile* [180]. Thus, by repressing σ^D^ transcription in the presence of high c-di-GMP levels, the Cdi1_3 riboswitch indirectly represses the synthesis of TcdA and TcdB.

Another c-di-GMP-responsive riboswitch, Cdi2_2, precedes the genes encoding the signal transduction system CmrRST in *C. difficile*, where CmrS is a putative histidine kinase, and CmrR and CmrT are two predicted response regulators [181]. Binding of c-di-GMP to Cdi2_2 positively affects *cmrRST* expression [182], most likely through anti-termination of transcription. Interestingly, *cmrRST* is also regulated by phase variation with an invertible DNA sequence, the cmr switch, located upstream of the corresponding genes [182]. CmrRST modulates colony morphology (rough or smooth) with the activation of *cmrRST* expression by c-di-GMP binding to Cdi2_2, promoting the development of rough colonies. CmrRST also inversely regulates motility and surface migration and promotes bacterial chaining. Importantly, a *cmrR* deletion mutant is unable to colonize the intestinal tract and has a strong virulence defect in the hamster model of infection [182].

In *Enterococcus faecalis* and *L. monocytogenes*, a riboswitch responding to vitamin B_12_ controls the transcription of *trans*-acting sRNAs EutX and Rli55, respectively, which both sequester the EutV response regulator of the EutVW TCS [183,184]. In these two pathogens, ethanolamine is sensed by the sensor kinase EutW, which results in the activation of EutV. EutV is a member of the ANTAR (AmiR and NasR transcriptional antiterminator regulators) family of response regulators that binds RNA rather than DNA and controls gene expression through transcription antitermination [185]. Specifically, EutV binds ANTAR elements in the 5’ UTR of *eut* mRNAs, whose products enable ethanolamine utilization, preventing the formation of a transcription terminator to activate *eut* expression. However, an ANTAR binding site is also present within sRNAs EutX and Rli55. Thus, when vitamin B_12_, a cofactor required for ethanolamine catabolism, is absent, EutX and Rli55 are synthesized and sequester the activated EutV to inhibit the expression of the *eut* genes. In contrast, when present, vitamin B_12_ binds to the riboswitch, which leads to premature termination of transcription and prevents the synthesis of the sRNAs. Consequently, EutV is not sequestered and *eut* expression is activated. Importantly, constitutive expression of EutV sRNA in a riboswitch deletion mutant significantly attenuates *L. monocytogenes* virulence in a mouse intravenous infection model [184].

### 2.5. Other TCS-Associated sRNAs Involved in Virulence

From numerous examples covered in this review, it is evident that TCSs in concert with sRNAs play a crucial role in virulence control in major pathogens. Recent studies described in this part illustrate the TCS-dependent control of virulence factor gene expression that is mediated by sRNAs in *Streptococcus*.

In the group A *Streptococcus* (GAS), *fasX* encodes an sRNA regulated by the FasBCA system, which comprises two sensor histidine kinases (FasBC) and one response regulator (FasA) [89,186]. The 205 nt *fasX* is the fourth gene of the *fasBCAX* locus and the transcription of this sRNA is growth phase-dependent with the higher expression levels occurring during the transition between the exponential and stationary phases of growth [89,187]. However, the specific signals leading to the activation of FasBCA remain to be defined. FasX has both positive and negative regulatory targets and was proposed to function as a master regulator governing the transition between colonization and dissemination [186]. On the one hand, FasX inhibits the translation of pili mRNAs and of *prtF1* and *prtF2* encoding fibronectin-binding proteins by base-pairing to sites overlapping the respective RBS [88,186,188]. Interestingly, FasX-mediated regulation of pilus genes occurs in a serotype-specific manner with FasX interacting with different mRNA sequences or different mRNA pilus biosynthesis genes in each serotype [188]. On the other hand, FasX positively affects the production of the thrombolytic agent streptokinase by base-pairing to the 5’ UTR of *ska* mRNA and forming a secondary structure, which enhances the stability of the mRNA [88,89,188]. FasX was shown to reduce GAS adherence and promote virulence in a bacteremia model using human plasminogen-expressing mice [88,188].

The TCS CovRS, a major global regulator of GAS virulence, directly or indirectly represses transcription of approximately 15% of the GAS genes [189]. Direct targets of CovR include the *riv* operon genes *rivR* and *rivX*, encoding respectively a transcriptional regulator and an sRNA [92,190]. The function of RivR and RivX remains controversial. RivR and RivX were first shown to positively regulate in an independent manner the expression of regulon members of the virulence gene regulator Mga [92]. However, these data were not corroborated in a more recent report and no regulatory functions could be attributed to RivX [191]. In addition, the virulence of a *rivRX* deletion mutant is attenuated [92,191], but the specific contribution of RivX to this phenotype is unclear.

## 3. Participation of sRNAs in Regulatory Networks

As illustrated in this review, TCSs in concert with sRNAs allow pathogenic bacteria to quickly sense and respond to different stimuli during host colonization. TCSs and stress-related sigma factors, regulatory RNAs and their protein partners constitute the major components of mixed regulatory networks to tightly control gene expression (Figure 1). It is important to emphasize that in many cases the expression of sRNA genes is controlled by TCSs (Table 1) and alternative sigma factors establishing a link between environmental conditions and downstream sRNA-mediated regulations. sRNAs in turn are involved in the control of gene expression for other regulatory components including sometimes their own transcriptional regulator or a different system. Such examples of feedback regulatory loops on sRNAs controlling TCSs are highlighted in Table 1. We have selected recent studies notably on sRNAs in Gram-negative and Gram-positive bacteria. The most extensively studied case of interconnection between TCS and sRNAs is OmrA and OmrB sRNAs regulated by OmpR-EnvZ TCS, which is in turn controlled by the same sRNAs [32]. Remarkably, the regulons of different TCSs usually contain several regulatory RNAs, leading to complex overlapping circuits allowing better integration of external signals. Intriguingly, in many cases, several homologous sRNAs are implicated in TCS signaling pathways with additive (e.g., *V. harveyi* Qrr1-5) or redundant (e.g., *E. coli* CsrB-C, *V. cholerae* Qrr1-4) action [14]. TCSs have been identified as targets for a number of sRNAs for which TCS-dependent regulation has not been uncovered. This connection allows to expand the regulatory potential of sRNA including associated TCS regulon. Specific examples of sRNAs targeting TCS genes are presented in Table 2. Such regulatory connections have recently been described in both Gram-negative and Gram-positive pathogens and are associated with stress response, colony morphology, motility, competence control, and other virulence-related processes [13,192].

The advantages of combining sRNA-mediated regulation with protein-mediated control are multiple. One of the first arguments in favor of RNA-based regulations is that sRNAs can be rapidly synthetized, requiring less cell resources compared to the protein, allowing them to ensure a rapid response to an external signal [13]. Their relatively short half-lives also allow a fast recovery after removal of the stimulus. This may explain why they are an advantage in fast adaptive responses to different stimuli [197]. Another advantage to include sRNAs in regulatory networks is their ability to cooperate with the protein regulatory factors acting at different regulatory levels [198]. For example, while the protein repressor inhibits the transcription by binding the specific DNA element, the sRNA contributes to further repression on post-transcriptional level by base-pairing the mRNAs still present in the cell. This cooperation is especially effective when the mRNA has a long half-life [198]. The participation of sRNA in regulatory circuits thus enables fine-tuning of target gene expression [198].

Like transcriptional regulatory proteins, sRNAs, in particular the *trans*-encoded sRNAs, often have multiple targets [199]. The regulation of a specific target with respect to another depends on the amount and ratio between the regulatory RNAs and target mRNA, as well as the affinity that the sRNA has for the mRNA. This results in a hierarchical regulation depending on the specific induction conditions [198]. It is important to consider that the sRNA can be degraded together with the target within a duplex, impacting the whole regulation network [200]. Moreover, especially in Gram-negative bacteria, the competition between different sRNAs for RNA-binding proteins like Hfq may alter their regulatory function [201]. sRNA can also be important actors in the cross talk between specific signaling networks. For example, MicA is able to repress the operon encoding PhoPQ TCS probably to repress the possible upregulation of OMPs during stress condition [195]. Finally, one of the specific aspects of regulation mediated by sRNA is the evolvability: new sRNA can quickly emerge and targets can be acquired or lost [13]. This characteristic allows them to rapidly adapt for a specific group of organisms in an environmental niche [13].

## 4. Strategies to Identify TCS-Associated sRNAs

### 4.1. Global Approaches for sRNA Identification

A combination of bioinformatics and experimental genomic approaches is largely used for the identification of sRNAs and their characterization. Integration of RNA-seq data with computational analysis led to the robust identification of a great number of potential sRNAs in many bacterial species (reviewed in [202,203,204]). Global approaches provide rich data requiring further targeted experimental validations for selected number of candidates. Northern blot and quantitative RT-PCR allow detection of new sRNAs and their expression profile analysis. In vitro gel mobility shift assays, sRNA-mRNA duplex stability and processing analysis, toeprinting assays, as well as plasmid-based reporter assays and compensatory mutational analysis are among powerful tools for sRNA target validation and mechanistic studies of sRNA-dependent control of target gene expression (reviewed in [205]).

Several recent reviews describe in detail the diversity of RNA-sequencing technologies developed for different aspects of regulatory RNA analysis [203,206]. Transcriptional start site (TSS) mapping by differential RNA-seq (dRNA-seq) or 5’-end RNA-seq, whole-transcript RNA-seq, small RNA sequencing (sRNA-seq), and Term-seq for 3’-end RNA mapping could be cited among the most powerful unbiased approaches for genome-wide sRNA identification [207,208,209]. These deep sequencing approaches have been successfully implemented for sRNA discovery in Gram-negative and Gram-positive bacterial pathogens including *Helicobacter pylori* [207], *L. monocytogenes* [210,211], *P. aeruginosa* [212,213], *L. pneumophila* [214], *Agrobacterium tumefaciens* [215], *Mycobacterium tuberculosis* [216], *C. difficile* [177], *Corynebacterium glutamicum*, *C. jejuni* [217], *Agrobacterium fabrum* [218], *Streptococcus agalactiae* [219], *V. cholerae* [220], *S. aureus* [221], *E. faecalis* [211], *N. meningitidis* [222], *Leptospira interrogans* [223], *Acinetobacter baumannii* [224], *Streptococcus pyogenes* [225], and *S. pneumoniae* [226].

The recently developed transcriptomic approach of dual RNA-seq is particularly suitable for the discovery of sRNAs contributing to virulence control in pathogenic bacteria [41]. Dual RNA-seq allows to simultaneously monitor the induction of host responses to infection and the bacterial sRNAs that shape the interactions of the pathogen with its host. This approach was originally developed for the analysis of cells infected with intracellular bacteria but has also been used more recently for extracellular pathogens. The application of dual RNA-seq to *Salmonella* infection of human cells identified the previously uncharacterized PinT as a highly up-regulated sRNA during infection within macrophages that serves as a timer for switching between the virulence programs of this pathogen [41].

Application of RNA-seq technologies to analyze bacterial secRNome provides promising perspectives to expand the role of sRNAs in host–pathogen interactions [227]. Secreted RNAs and RNAs in extracellular vesicles (membrane vesicles and OMVs) with potential regulatory function in host could be characterized in both Gram-negative and Gram-positive bacteria, including recent examples in *P. aeruginosa* and *L. monocytogenes* [79,228]. Interestingly, Rli32, one of these sRNAs that have been identified as potential inducers of type I interferons IFN response during *L. monocytogenes* infection is part of the VirR/VirS and Agr quorum sensing regulons [79,229].

Many sRNAs act in concert with protein partners such as the RNA chaperone proteins Hfq and ProQ, facilitating base-pairing of sRNAs with their targets [230] or ribonucleases mediating their regulatory actions [231]. A specific class of sRNA regulators functions through sequestration of a regulatory protein such as the CsrA pleiotropic regulator [232]. Global identification of RNA ligands associated with RNA-binding proteins represents another largely used genomic strategy for sRNA discovery (reviewed in [203]). These global approaches include RNA immunoprecipitation followed by RNA-seq (RIP-seq) or (CLIP-seq) including an UV cross-linking step to covalently link protein–RNA interactions, RNA Interaction by Ligation and sequencing (RIL-seq), Cross-linking, Ligation, and Sequencing of Hybrids (CLASH) or Gradient profiling by sequencing (Grad-seq). When these approaches include an RNA-RNA ligation step, they lead to sRNAs direct target identification (see below).

*In silico* analyses help to predict both new sRNAs and potential sRNA targets. Bioinformatics predictions of new sRNAs are generally based on comparative genomics, RNA secondary structure analysis, and in silico searches for promoters and Rho-independent terminators associated with intergenic regions or located in antisense orientation to annotated genes in bacterial genomes [233]. Several programs are available for sRNA target predictions including TargetRNA, IntaRNA, CopraRNA, sTarPicker, RNAhybrid, and RNADuplex that can be used individually or in combination (reviewed in [234,235]).

Experimental validation of in silico predicted sRNA targets is required since these bioinformatics analyses usually lead to a large number of potential targets with a substantial proportion of false positive predictions [234]. Direct sRNA target identification still remains a challenging task, but several new experimental approaches have been recently developed for unbiased sRNA target discovery (reviewed in [206]). Among them, MS2-affinity purification coupled with RNA sequencing (MAPS) [236,237] allows sensitive detection of direct sRNA targets by base-pairing with an sRNA of interest, while RIL-seq [238] and CLASH [239] are used for global sRNA–RNA interaction analysis and rely on the association of sRNA-target pairs with RNA-binding proteins.

### 4.2. Specific Approaches Adapted for TCS-Associated sRNAs

Specific strategies have also been implemented for the identification of sRNAs related to TCS. In silico approaches are valuable for the prediction of TCS regulators involved in the regulation of sRNA expression. For example, an in silico search for the presence of a consensus sequence recognized by the specific response regulator CiaR identified several sRNAs genes as members of the CiaRH TCS regulon in *S. pneumoniae* [161]. A further study combined the sRNA target prediction with experimental validations to define the CiaRH-dependent csRNA targets [171]. Functional enrichment represents a powerful computational approach to specify the pathways targeted by sRNAs. Interestingly, such approach placed TCS as an enriched category of genes targeted by *cis*-encoded sRNAs in *M. tuberculosis* [216].

In *C. perfringens*, new members of the VirR/VirS TCS regulon have been discovered by a differential display method [97]. Screening of 1200 plasmids from a random chromosome library with cDNA probes from a *virR* mutant and wild-type strain led to the identification of VR-RNA as a secondary regulator in the VirR/VirS-dependent cascade controlling toxin production in this pathogen [94,95,97]. Two additional regulatory RNAs, VirT and VirU, were later discovered as direct VirR targets by in silico searches in *C. perfringens* genome sequence for the presence of the VirR box motif [240].

Global transcriptome analysis remains a tool of choice to define the regulons of transcriptional regulators [241]. Its application to comparative analysis of mutants inactivated for TCS response regulators can be combined with direct target identification by in silico or experimental chromatin immunoprecipitation followed by high-throughput sequencing (ChIP-seq) approaches to specify the regulatory cascades related to a given TCS. Integration of ChIP-seq results with RNA-seq data led to the discovery of the Mcr7 sRNA as a major PhoP target in *M. tuberculosis*, thus establishing a link between the PhoPR TCS, essential for virulence in this pathogen, and the downstream functions necessary for successful infection of the host [80]. Mcr7 was shown to modulate the Twin Arginine Translocation (Tat) protein secretion system involved in secretion of the immunodominant antigen Ag85 complex and the BlaC beta-lactamase in *M. tuberculosis*.

To broaden sRNA identification in *S. pneumoniae*, mutants of genes encoding the response regulator of three major TCSs: GRR, CbpR, and VncR have been analyzed by RNA-seq of small transcript-enriched fractions [242]. This study also used global pathogenesis profiling by Tn-seq focusing on the relative fitness level of sRNAs mutants in different infection sites in the host during infection to evaluate the contribution of sRNAs to pathogenesis. Transposon mutagenesis can also be used to discover new regulators of a given gene. For example, a forward genetic screen using a reporter gene fusion led to the identification of the KinB-AlgB TCS controlling CRISPR-Cas in *P. aeruginosa* [64].

The sequence-specific target recognition by sRNA of various functional classes involves a so-called “seed” region that is generally conserved among functional homologs of riboregulators [5]. The seed region is usually defined by sequence conservation analysis and mutational exchange for functional relevance. A novel sRNA-based approach has recently been developed to test the in vivo relevance of the conserved seed sequence under stress conditions [121]. Such seed sequences have been identified in ESR-regulated RNAs, RybB in *E. coli* and its functional homologues MicV and VrrA in *V. cholerae*. A library of synthetic sRNA regulators with randomized base-pairing region was first constructed and laboratory selection experiments for sRNA variants providing improved stress resistance were then performed. High-throughput sequencing of selected variants revealed that the seed-pairing domain of ESR-regulated sRNAs is strongly enriched among sRNAs identified under membrane-damaging conditions [121]. This strategy could be used for screening of complex bacterial phenotypes by using synthetic sRNA libraries and for the identification of the most efficient sRNA sequences for gene expression control and further applications.

## 5. Antimicrobial Strategies Related with TCS and Associated sRNAs

Broad-spectrum antibiotics have revolutionized the medicine and saved many lives. While they are still indispensable drugs today to fight against bacterial infections, important problems arose from their massive use. One of the direct consequences is the spreading of multiple drug-resistant (MDR) bacteria, such as methicillin-resistant *S. aureus,* that represent a critical problem for public health [243,244]. Moreover, the perturbation in the microbial composition of human microbiota caused by broad-spectrum antibiotics is associated with many diseases [245]. Another aspect to consider is that infections caused by major pathogens are difficult to treat with conventional antibiotics because of specific resistance mechanisms related with their infection life cycle. This includes, in the case of *C. difficile,* the differentiation into spores, which are intrinsically resistant to antibiotics [180], or the acquisition of antibiotic resistances through HGT in many pathogens [246,247]. There is therefore an urgent need to broaden the repertoire of new antibiotic targets and to find species-specific antimicrobial strategies. Because of their important roles in regulating virulence factors and genes for antibiotic resistance in major human pathogens, TCSs and sRNA-regulated transcripts are ascendant targets for new antimicrobial strategies. In higher eukaryotes, the signal transduction systems are generally based on serine, threonine and tyrosine phosphorylation, while the bacterial signaling systems imply histidine phosphorylation. These differences encourage the research on selective inhibitors of bacterial TCS [248,249]. For example, the small molecule LED209, identified by high-throughput screenings, inhibits the activation of the HK component QseC, comprised in the QseCB TCS, by AI-3 in different Gram-negative pathogenic bacteria. This inhibition impacts bacterial pathogenicity in vitro and in vivo but not cell growth [250]. Because of their function to sense the host conditions, TCSs together with transcriptional regulators are central actors in the design of engineered probiotics [251,252]. As a proof of concept to treat bacterial infection, the probiotic *E. coli* Nissle has been engineered to sense tetrathionate, a molecule produced in the inflamed gut during *Salmonella* infection, through the TtrS/R TCS. In response to this signal, *E. coli* secretes an antimicrobial peptide, Microcin H47, that inhibits *Salmonella* growth [253].

Moreover, the ability of sRNAs to silence gene expression has led to the development of a new antimicrobial strategy based on short antisense oligonucleotides (ASOs). The RNA-targeted therapeutics in the form of ASOs are a seductive treatment to cure human diseases including bacterial infections. While commercialized antisense therapy to treat diseases such as Cytomegalovirus (CMV) Retinitis, Homozygous Familial Hypercholesterolaemia (HoFH) and Spinal muscular atrophy (SMA) (complete list in review [254]) already exist, there are no approved RNA-targeted antimicrobials to cure bacterial infections. With the characterization of novel sRNAs important for the pathogenesis in bacteria, this strategy seems more plausible in the future. The increasing knowledge on the molecular mechanisms of natural *trans*-encoded RNA action in gene silencing by base-pairing led to a design of antisense antimicrobial therapeutics able to silence expression of bacterial essential genes [255]. To treat the skin infections caused by *S. aureus,* the ASO called CPP-PMO has been designed to target the mRNA of the essential gene *gyrA.* When administrated ectopically on skin wound in mice, the CPP-PMO reduces the viability of *S. aureus* [256]. Targeting virulence genes with ASO is an appealing approach to develop a new class of antimicrobials especially for MDR bacteria. Converting MDR bacteria to drug hyper susceptible cells is a potential approach to fight against MDR infection. It has been recently shown that the overexpression of the sRNA AS1974 in clinical isolates of MDR *P. aeruginosa* induces drug hyper susceptibility [257].

Beside the tempting perspective of developing RNA-targeted ASOs as new species-specific antibiotics, three main concerns derive from the use of RNA. First, RNA molecules are too unstable to be administrated as drugs. Nevertheless, existing chemical classes of modified nucleic acids with improved stability and nuclease resistance may be used to tackle this issue [255]. Second, the uptake of such molecules is difficult, so the ASOs are usually short and carry a cell-penetrating peptide (CPP) [258]. Third, ASOs usually sequester the RBS of an mRNA. Because RBS is a low complexity zone, off-targeting effects may be observed in other bacteria with similar RBS, thus compromising the killing specificity [259]. Rapidly accumulating knowledge on the “seed” regions as essential determinants for specific recognition by sRNAs of their targets could help to increase the specificity of programmable RNA-based antimicrobials [5]. New ideas have recently emerged suggesting the use of genes regulated by the sRNA, regulatory RNAs themselves and their protein interaction partners (e.g., Hfq or CsrA), as novel drug targets due to their contribution to antibiotics response and resistance (see reviews [131,260,261]). Interestingly, an inhibitor peptide (RI20) of Hfq-sRNA interactions has been identified in *E. coli* that inhibits Hfq function, increasing the susceptibility of *E. coli* to antibiotics [262].

Retargeting CRISPR-Cas system against pathogens has a great potential for development of programmable antimicrobials. CRISPR RNAs could be easily designed to target specific sequences in genes related to virulence of pathogenic strains. Delivery of engineered CRISPR-Cas system components using phages or phagemids has been suggested as an interesting therapeutic perspective for selective removal of bacterial pathogens and precise microbiome composition alteration [263]. On the other hand, endogenous CRISPR-Cas systems represent an important obstacle for development of alternative antimicrobial strategies based on phages. The discovery of quorum sensing control of CRISPR-Cas adaptive immunity system opens new therapeutic perspectives to suppress CRISPR function for medical applications combining phage therapy with quorum-sensing inhibitors [158,264].

Riboswitches have recently emerged as possible targets for the development of alternative antimicrobial approaches (reviewed in [265,266]). In *C. difficile*, a great number of riboswitches has been identified [9,177], including c-di-GMP-responsive riboswitches important for virulence control [267,268]. Widespread use of riboswitches to control essential metabolic pathways motivated the research of ligand analogs with bactericidal activity. For example, a potential of guanine analogs targeting guanine-sensing riboswitches to inhibit *C. difficile* growth has been evaluated [269]. Similar strategies appear promising for the development of antimicrobials against *S. aureus* and other Gram-positive bacteria [270,271]. An efficient protection against *C. difficile* infection has recently been reported in mice for a riboflavin analog binding flavin mononucleotide FMN riboswitch without inhibiting normal cecal flora [272].

Another emerging application of sRNAs is their ability to be secreted in the extracellular space to modulate the immunity of the host [227]. Future characterization of secRNome of major pathogens will shed a new light on the role of sRNAs in host–pathogen interactions. The recent discovery of Rli32 sRNA inducing interferon response during *L. monocytogenes* infection illustrates well this yet unexplored regulatory potential [79]. This new field of research could suggest new ways to transport RNA inside the host and new possibilities for the modulation of host immune system.

## 6. Conclusions

Regulatory sRNAs have recently emerged as important components of regulatory networks controlling gene expression in bacterial pathogens. New technological developments provided powerful tools to study these regulatory processes. Multiple interconnections with signal transduction pathways have been revealed that ensure adequate responses to changing environments and survival of pathogens inside the host. New findings in this emerging field of RNA-based host–pathogen crosstalk could be anticipated in the near future. Accumulating knowledge on the molecular mechanisms involved in these adaptive responses and interactions of pathogens with their hosts paves the way for the development of new antimicrobial strategies as a challenging task for coming years. Deciphering molecular details of RNA-based control of gene expression in bacterial pathogens constitutes an essential step for harnessing these yet poorly explored powerful mechanisms in the future for specific therapeutic and epidemiological applications.

## Figures and Tables

**Table 1:** Examples of TCS-associated sRNAs in Gram-negative and Gram-positive bacterial pathogens.

Pathogen	TCS		sRNA Regulated by TCS ^a^	Targets ^b^	Roles in Virulence	Stimuli	References
	HK	RR					
**Gram-negative bacteria**							
*Escherichia coli*, *Salmonella enterica* serovar Typhimurium	CpxA	CpxR	(+) CpxQ (−) CyaR (+) RprA	CpxQ: *nhaB* (Na-H antiporter), *skp* (chaperone protein), *agp* (periplasmic acid glucose-1-phosphatase), *fimA* (major fimbrin subunit of type 1 pilus) and *ydjN* (cystine transporter); CyaR: *ompX* (outer membrane protein), *luxS* (autoinducer-2 synthase), *nadE* (essential NAD synthetase) and *yqaE* (membrane protein with unknown function), *hdeD* (acid-resistance membrane protein); RprA (see below);	Envelope-stress response	High pH, osmolality, alteration in IM lipid composition	[25,26,27,28,29,30]
	EnvZ	OmpR	(+) OmrA (+) OmrB (+) MicF	OmrA/OmrB: *ompT* (outer membrane protease), *cirA*, *fecA*, *fepA*, (receptors for iron–siderophore complexes), *csgD* (TR of curli genes), *flhDC* (master regulator of flagellar synthesis) and *ompR-envZ* (TCS); MicF: *ompF* (outer membrane protein), *cpxA-cpxR* (TCS), *lrp* (TR);	Envelope-stress response	Osmolality	[31,32,33,34,35,36,37]
	PhoQ	PhoP	(+) MgrR, (+) AmgR (in *Salmonella*) (+) PinT (in *Salmonella*)	MgrR: *eptB* (phosphoethanolamine transferase), *ygdQ* (hypothetical protein) and *soxS* (TR involved in oxidative stress); AmgR: *mgtC* (virulence protein); PinT: *hilA* (hyperinvasion locus A) and *rtsA* (TR)	Resistance to CAMPs, survival within macrophages, expression of type 3 secretion system (T3SS)	Low Mg2+/Ca2+, antimicrobial peptides	[38,39,40,41,42,43]
	RcsC RcsD ^c^	RcsB	(+) RprA	*rpoS* (central regulator of the general stress response), *csgD* (TR of curli genes) in *S.* Typhimurium, *hdeD* (acid-resistance membrane protein);	Inhibition of biofilm development and stress response	Alterations of bacterial envelope	[30,44,45,46]
	GlrK (QseE YfhK)	GlrR (QseF, YfhA)	(+) GlmY and GlmZ	GlmY: GlmZ; GlmZ: *glmS* (glucosamine-6-phosphate synthase), *Locus of Enterocyte Effacement (LEE) 4* and *LEE5* operons, *espFu* and *nleA* (non-LEE-encoded effectors)	Antibiotic resistance, attaching and effacing (AE) lesion formations	Host hormones such as epinephrine and norepinephrine in Enterohemorrhagic *E. coli* (EHEC)	[47,48,49,50]
	QseC	QseB	(+) GlmY			Autoinducer 3 (AI-3) and the adrenergic hormones	[51]
	ArcB	ArcA	(−) ArcZ	*rpoS* (central regulator of the general stress response), *agrB-agrA* (TCS), *flhDC* (master regulator of flagellar genes);	Regulation of motility, host adaptation	Aerobic conditions	[34,52]
*Helicobacter pylori*	ArsS	ArsR	*Cis*-antisense 5′*ureB*-sRNA	*ureAB* (urease)	Colonization of the gastric mucosa	Low pH	[53]
*Vibrio cholerae*	LuxPQ ^d^	Lux O	(+) Qrr1-4	*hapR* (master TR), large type 6 secretion system cluster	Expression of virulence and biofilm genes	Cell density—AI2	[54,55,56,57]
	CqsS	LuxO					
	EnvZ	OmpR	(+) CoaR	*tcpI* (TR)	Expression of major pilin subunit	Osmolality	[58,59]
*Pseudomonas aeruginosa*	CbrA	CbrB	(+)CrcZ	Sequestration of the RNA-binding protein Crc	Metabolism, susceptibility to antibiotics and virulence	Carbon and nitrogen source	[60,61]
	NtrB	NtrC	(+)NrsZ	*rhlA* (rhamnolipid biosynthesis)	Increases production of the virulence factor rhamnolipid	Nitrogen limitation	[62]
	NarX	NarL	(+)PaiI	Unknown	Colonization of tumor	Low oxygen and nitrate	[63]
	KinB	AlgB	Type I CRISPR-Cas		Immunity to phage infection	Unknown	[64]
	PmrA	PmrB	(+) RsmY	Sequestration of the RNA-binding protein RsmA	Indirect effect on the expression of *lipA* (lipase)	Low Mg ^2+^	[65]
TCS BarA-SirA and its homologous systems							
*S* *. typhimurium*	BarA	SirA	(+) CsrB (+) CsrC	Sequestration of the RNA-binding protein CsrA/RsmA	Metabolism, motility, biofilm formation, stress resistance, virulence and quorum sensing	BarA senses the presence of carboxylate compounds.	[54,66,67,68,69,70,71]
*E. coli*	BarA	UvrY					
*P. aeruginosa*	GacS	GacA	(+) RsmY (+) RsmZ				
*Legionella pneumophila*	LetS	LetA	(+) RsmY (+) RsmZ				
*V. cholerae*	VarS	VarA	(+) CsrB (+) CsrC (+) CsrD				
**Gram-Positive Bacteria**							
*Listeria monocytogenes*	LisK	LisR	(+) LhrC 1–5	*lapB* (virulence adhesin), *tcsA* (T cell-stimulating antigen), *oppA* (oligo-peptide binding protein)	Adhesion and invasion of non-phagocytic cells, expression of T cell-stimulating antigen TcsA	Cell envelope stress	[72,73,74,75,76]
	LisK	LisR	(+) Rli22	*oppA* (oligo-peptide binding protein)	Expression of virulence-associated oligo-peptide binding protein	Cell envelope stress	[77]
	VirS	VirR	(+) Rli32	Inducer of IFN-β expression; *lhrC* locus (LhrC1-5)	Induction of IFN-β expression	In vivo infection	[78,79]
*Mycobacterium tuberculosis*	PhoR	PhoP	(+) Mcr7	*tatC* (Twin Arginine Translocation (Tat) protein secretion apparatus)	Secretion of the immunodominant Ag85 complex and the beta-lactamase BlaC	Unknown	[80]
*Staphylococcus aureus*	AgrC	AgrA	(+) RNAIII (+) ArtR	RNAIII: *rot*, *mgrA* (master TRs); *spa, coa, sbi, Sa1000, lytM, hla* (virulence factors); ArtR: *sarT* (TR)	Evasion of host immunity, toxins expression	Cell density (autoinducers molecule)	[81,82]
					α-toxin expression	Cell density	[83]
	SsrB	SsrA	(+) RsaE	*opp3A* (ABC transporter component) *rocF* (arginase involved in the arginine catabolism). In *Staphylococcus epidermidis. lrgA* (antiholin) *icaR* (*icaADBC* biofilm operon repressor) and *sucCD* (succinyl-CoA synthetase)	Metabolic adaptation, biofilm formation, eDNA release.	Low O_2_ and NO	[84,85,86,87]
Group A *Streptococcus*	FasB/FasC	FasA	(+) FasX	*fasBCA* (TCS), *ska* (streptokinase), pilus operon, *cpa* (minor pilin protein).	Adhesion, motility and adherence	Unknown	[88,89,90]
	CiaH	CiaR	(+) 5 csRNAs	*comC* (precursor of the competence stimulating peptide)	Competence, genomic plasticity	Unknown	[91]
	CovS	CovR	(+) RivX	possibly *mga* (master TR)	Mga regulates genes important for virulence in the host	Unknown	[92]
*Clostridium perfringens*	VirS	VirR	(+) VR-RNA VirT, VirU	VR-RNA: *plc* (Phospholipase C), *colA* (κ-toxin)	Expression of toxins		[93,94,95,96,97]

^a^ TCSs may control the expression of the sRNA in a positive (+) or negative (−) way. ^b^ The sRNA name is specified when multiple sRNAs are regulated by the TCS. TCSs as sRNA targets are in bold. TR: Transcriptional Regulator. ^c^ The Rcs system is a non-ortholog TCS composed of the transmembrane sensor kinase RcsC, the transmembrane protein RcsD, and the response regulator RcsB. ^d^ AI-2 is detected by the periplasmic protein LuxP in complex with the LuxQ histidine kinase. Phosphate is then transduced to a single phosphotransfer protein, LuxU, which in turn transfers the phosphate to a response regulator called LuxO.

**Table 2:** Examples of sRNAs regulating TCSs.

Pathogen	sRNA Regulating TCS	Regulated TCS	References
**Gram-negative Bacteria**			
*Escherichia coli*	RybC	DpiA-DpiB	[193]
*E. coli*	SdsN	NarQ-NarP	[194]
*E. coli*, *Salmonella*	GcvB, MicA	PhoP-PhoQ	[195,196]
**Gram-positive Bacteria**			
*Staphylococcus aureus*	psm-mec	AgrA-AgrC	[150]
*Clostridioides difficile*	Cd2-2	CmrR-CmrS-CmrT	[182]
*Streptococcus pneumoniae*	srn206	ComD-ComE	[170]
*Enterococcus faecalis*, *Listeria monocytogenes*	EutX, Rli55	EutVW	[183,184]
